# Glioblastoma update: molecular biology, diagnosis, treatment, response assessment, and translational clinical trials

**DOI:** 10.12688/f1000research.11493.1

**Published:** 2017-10-26

**Authors:** Frank Lieberman

**Affiliations:** 1 Neurooncology Program, UPMC Hillman Cancer Center, UPMC Cancer Pavilion, Pittsburgh, PA, USA

**Keywords:** glioblastoma, molecular pathology, neuroimaging, immunotherapy, pseudoprogression, clinical trials

## Abstract

This is an exciting time in neuro-oncology. Discoveries elucidating the molecular mechanisms of oncogenesis and the molecular subtypes of glioblastoma multiforme (GBM) have led to new diagnostic and classification schemes with more prognostic power than histology alone. Molecular profiling has become part of the standard neuropathological evaluation of GBM. Chemoradiation followed by adjuvant temozolomide remains the standard therapy for newly diagnosed GBM, but survival remains unsatisfactory. Patients with recurrent GBM continue to have a dismal prognosis, but neuro-oncology centers with active clinical trial programs are seeing a small but increasing cadre of patients with longer survival. Molecularly targeted therapeutics, personalized therapy based on molecular profiling of individual tumors, and immunotherapeutic strategies are all being evaluated and refined in clinical trials. Understanding of the molecular mechanisms of tumor-mediated immunosuppression, and specifically interactions between tumor cells and immune effector cells in the tumor microenvironment, has led to a new generation of immunotherapies, including vaccine and immunomodulatory strategies as well as T-cell-based treatments. Molecularly targeted therapies, chemoradiation, immunotherapies, and anti-angiogenic therapies have created the need to develop more reliable neuroimaging criteria for differentiating the effects of therapy from tumor progression and changes in blood–brain barrier physiology from treatment response. Translational clinical trials for patients with GBM now incorporate quantitative imaging using both magnetic resonance imaging and positron emission tomography techniques. This update presents a summary of the current standards for therapy for newly diagnosed and recurrent GBM and highlights promising translational research.

## Introduction

The second decade of the 21 ^st^ century is an exciting time in neuro-oncology, perhaps especially so in the diagnosis and management of glioblastoma multiforme (GBM). The results of ongoing discovery related to the molecular genetics of GBM, the molecular pathways mediating resistance to immunotherapy, and deeper understanding of mechanisms of sensitivity and resistance to molecularly targeted agents are all entering translational clinical trials. In addition, innovative approaches to the neuroimaging of treatment response are improving our ability to differentiate between effects of therapy and tumor progression in both newly diagnosed and recurrent GBM.

## Discussion

Approximately 70,000 primary CNS tumors are diagnosed annually in the US, with GBM being the most frequent high-grade glioma, with an incidence of 3–4/100,000 ^1^. The incidence increases with age, with the peak incidence being in the fifth or sixth decade. Although there have been a number of advances in the therapy of GBM, median survival is still short at between 15 and 18 months for patients with newly diagnosed GBM, approximately 10% going on to be five-year survivors ^2^. The median survival for patients with recurrent GBM treated with regimens including bevacizumab is eight to nine months ^1^. Neuro-oncologists, being optimists by nature, note that long-term survivors are being seen with increasing frequency, and the median survival has been improving incrementally over the 12 years since the original report of the efficacy of chemoradiation incorporating temozolomide followed by adjuvant temozolomide for six monthly cycles was reported in 2004 ^3^.

Current treatment regimens for newly diagnosed GBM are based on the randomized prospective trial, led by Roger Stupp, comparing external beam fractionated radiation alone to concurrent daily low-dose temozolomide during radiation, a one-month break, and then six months of adjuvant temozolomide at 150–200 mg/m ^2^. This trial demonstrated an increase in median overall survival of approximately three months and 30% versus 10% survival at 24 months favoring the chemotherapy cohort ^2, 4^. This regimen has been the basic scaffold for subsequent trials, and studies of molecular prognostic and predictive factors are based on analysis patient cohorts treated with this regimen or permutations that maintain the basic design.

Although the introduction of anti-angiogenic therapies, with bevacizumab being the lead drug in class, initially appeared to be a transformative approach, subsequent clinical trial experience has been disappointing. Initial studies for recurrent GBM demonstrated that treatment with bevacizumab was associated with higher response rates, clinical improvement, and longer time to progression than historical controls employing chemotherapy ^5– 7^. Two randomized prospective trials in which bevacizumab was added to the standard chemoradiation followed by adjuvant temozolomide failed to demonstrate a benefit in overall survival for the bevacizumab arm ^8, 9^. Phase 2 trials evaluating bevacizumab in combination with cytotoxic chemotherapies subsequently failed to demonstrate a benefit in overall survival with the combinations compared to bevacizumab alone ^10^. In a randomized phase 2 trial comparing bevacizumab or lomustine alone to the combination of bevacizumab and lomustine with nine-month survival as the primary endpoint, the combination of bevacizumab and lomustine was superior to either of the drugs alone, but bevacizumab alone was no better than lomustine alone ^11, 12^. The EORTC trial 26101 compared the combination of bevacizumab and lomustine to lomustine alone and failed to demonstrate improved overall survival in the combination arm (Proceedings Society for Neurooncology, 2017). Currently, outside of clinical trials, the role of bevacizumab in the treatment of GBM is in treating patients with neurologic symptoms and signs related to the size of the tumor or the surrounding edema. This benchmark for overall survival with bevacizumab is only eight to nine months ^5, 13^. Although the addition of bevacizumab to chemoradiation and adjuvant temozolomide did not prolong overall survival, bevacizumab can ameliorate radiation-induced worsening of edema and mass effect in newly diagnosed GBM patients.

Discoveries in molecular neuropathology have demonstrated that, although essentially a homogeneous group of tumors by histologic criteria, GBM can be separated out into clinically relevant subgroups using molecular classification schemes ^14, 15^. Microarray studies performed as part of the Cancer Genome Project led to a four-compartment classification, separating GBM into classical, pro-neural, neural, and mesenchymal subgroups. Similar to risk prediction multigene panels used in other malignancies, retrospective molecular and outcome correlative studies involving patients treated with chemoradiation and temozolomide for newly diagnosed GBM identified a nine-gene panel which separates cohorts into those with better and worse prognosis ^16^. Genetic profiling appears to separate tumors which arise from pre-existing low-grade gliomas from those which arise primarily as GBM ^14, 15^. Mutations in the isocitrate dehydrogenase (IDH) gene, constituting one of the earliest and possibly initiating mutations in gliomas, are one discriminant between primary and secondary GBM ^17^. *IDH* has two isoforms, with mutation in *IDH1* (IDH1-R132H) being the most common. *IDH* mutations are present in 80% of secondary GBM but in only 5% of primary GBM. The presence of *IDH* mutation may identify a better prognostic subgroup within GBM patients. Approximately 40–50% of GBM have *EGFR* gene amplification, and a splice variant which creates a mutant form of EGFR (viii) is present in 20–50% of *EGFR* gene-amplified GBM ^14^. The prognostic implications of *EGFR* amplification are still unsettled; higher levels of gene amplification appear to correlate with poorer survival outcomes.

O6-methylguanine-DNA methyltransferase (MGMT) is involved in DNA repair of O06-alkylating agents, the prototypical example being temozolomide. MGMT promoter methylation has prognostic and predictive significance in patients with GBM, with longer survival rates in newly diagnosed patients treated with chemoradiation and subsequent adjuvant temozolomide ^14, 15^. MGMT promoter methylation is present in approximately 50% of newly diagnosed GBM but more commonly in secondary GBM. MGMT promoter methylation status has been shown to be a predictive biomarker for survival in elderly GBM patients. Epigenetic mechanisms also have prognostic significance in GBM. Tumors demonstrating hypermethylation of CpG sites throughout the genome, usually seen in younger patients with the pro-neural subtype, have an especially favorable prognosis ^14^.

Mutations in the telomerase reverse transcriptase gene ( *hTERT*) occur in approximately 75% of GBM cases. Although hTERT mutation status as a univariate factor does not appear to be prognostic or predictive for GBM treatment outcomes, the favorable prognosis conveyed by MGMT promoter methylation may depend on concurrent hTERT promoter mutation. Classification into subgroups based on MGMT promoter methylation status and hTERT promoter mutation status appears to be more robust than MGMT promoter methylation alone ^18, 19^. Since MGMT promoter methylation is a stratification factor in many clinical trials for GBM, hTERT mutation status will need to be correlated with outcome as well.

Retrospective analysis of molecular features and outcome in the NRG Oncology Trial RTOG 05-25, which tested two different regimens of adjuvant temozolomide, led to a proposed update for the prognostic recursive partitioning mode ^3^. This new model incorporates MGMT protein expression and c-MET protein expression with better separation of the overall survival prognostic groups than incorporating MGMT promoter methylation alone ^3^.

Translational clinical trial approaches for recurrent and newly diagnosed GBM include molecular targeted therapeutics, immunotherapies, and somatic gene therapy ^2^. Although trials of single-agent tyrosine kinase inhibitors for recurrent GBM have been uniformly disappointing, ongoing trials are applying insights into mechanisms of resistance and better understanding of driver mutations ^2, 20^. Current trial designs include detection of specific target mutations or mutational profiles as eligibility criteria for the specific targeted drug. GBM patients are able to participate in mutation-defined rather than histology-defined trials, such as the MATCH trial. Responses have been reported with molecular-targeted therapies for GBM expressing BRAFv600 mutations, for GBM expressing NF1 mutations ^21^, and for subsets of tumors with *EGFR* gene amplification. Although most studies with EGFR tyrosine kinase inhibitors (erlotinib and gefitinib) have been negative, retrospective molecular correlate studies of outcome suggest that a subgroup of tumors with EGFRviii mutation and wild-type *PTEN*, a tumor suppressor gene in the PI3K signaling pathway, do respond ^22, 23^. Clinical trials for recurrent GBM using a bifunctional antibody targeting EGFR and coupled to a microtubule-disrupting agent have been completed, and a study adding this agent to the standard chemoradiation and adjuvant temozolomide is ongoing (NCT02573324).

After a generation of persistent investigation by immunologists in the face of multiple negative trials, discoveries elucidating the mechanisms of tumor-induced immunosuppression in the tumor microenvironment have been translated into the clinic ^24, 25^. The first immunomodulatory drug trials in solid tumors have focused on the immunosuppressive signals PD1, PDL-1, CTLA4, and IDO. PD-1 inhibitors and CTLA-4 inhibitors have been FDA approved for melanoma and non-small-cell lung cancer trials. Clinical trials in recurrent and newly diagnosed GBM are 2–3 years behind other solid tumor trials, in part because of additional complexities of drug delivery within the tumor microenvironment ^26^. Nivolumab, pembrolizumab, and ipilimumab are humanized monoclonal antibodies with molecular weight and lipid/water solubility characteristics that likely limit penetration into the tumor microenvironment, especially in regions adjacent to tumor mass where the blood–brain barrier is relatively intact. Since PD-1 is expressed on T cells rather than tumor cells, targeting PD-1 may not require intratumoral drug delivery. Ongoing trials of checkpoint inhibitors in recurrent GBM have reported encouraging preliminary data suggesting activity ^27, 28^. However, in the Checkmate 143 trial, nivolumab did not meet the primary endpoint for overall survival compared to bevacizumab alone (Proceedings, World Federation of Neuro-Oncology Societies, 2017). Trials of checkpoint inhibitors, single drugs and combinations, are currently under phase 1 trial for newly diagnosed GBM.

Numerous studies of vaccines for recurrent and newly diagnosed GBM have been recently completed or are ongoing. A prospective randomized trial comparing temozolomide alone to temozolomide plus a vaccine targeting the EGFRviii-mutated protein demonstrated improved overall survival in the vaccine arm ^29^, but a similar trial in newly diagnosed GBM was terminated early after interim analysis indicated futility. The recurrent GBM EGFRviii trial was open label and compromised by a high rate of drop out in the standard treatment arm. Current vaccine strategies include autologous vaccines generated from the patient’s tumor at resection, peptide-based vaccines, and a new generation of vaccines using dendritic cells exposed to tumor cell RNA ^30, 31^. Viral vector-based gene therapy trials, in which the vector encodes immunomodulatory molecular signals, combine the lessons learned in viral somatic gene therapy trials to immunotherapy strategies ^32, 33^. Chimeric antigen receptor T cell therapies are also in early clinical trials for patients with GBM and have demonstrated the ability to migrate from injection sites to distant tumor sites ^34^.

A novel cytotoxic mechanism based on tumor treatment fields was the subject of a randomized prospective phase 3 trial in newly diagnosed GBM. This technology employs scalp electrodes to generate alternating directional fields of low-intensity radiation in the 150–200 kHz range through the tumor. The tumor treatment fields (TTFs) led to the disruption of mitotic spindle formation and cell death in dividing tumor cells ^35^. This trial demonstrated a survival benefit to the TTF cohort, similar in magnitude to the incremental benefit seen in the chemotherapy arm of the trial, which established concurrent chemoradiation and temozolomide as the standard therapy for newly diagnosed GBM ^4^. Controversy continues ^36^ regarding the lack of a sham control arm in the trial and whether the TTF arm was more compliant with temozolomide therapy, but analysis of the complete dataset indicates that the groups were well balanced for the relevant prognostic factors ^35^.

The increasing translational clinical trial focus on strategies different from cytotoxic chemotherapy has created challenges for the radiologic evaluation of treatment response and tumor progression ^37, 38^. Patients treated with chemoradiation for newly diagnosed GBM may have transient worsening in MRI findings manifest in the first post radiation therapy follow up MRI. This phenomenon, termed pseudoprogression, if not recognized can lead to premature discontinuation of effective therapy and is actually a good prognostic marker for prolonged survival ^38^. Pseudoprogression is not distinguishable from true progression using routine clinical MRI criteria. At present, no single imaging feature or combination of features have been validated as biomarkers differentiating pseudoprogression from true progression. Quantitative imaging techniques employing MR spectroscopy and data extracted from clinical sequences are supplementing and refining treatment response assessment based on visual inspection of images ^37^. Techniques that appear promising include Dynamic Contrast Enhanced or Susceptibility Weighted Contrast sequences, which allow the calculation of regional cerebral blood flow, changes in ADC characteristics, and PET imaging using FDG, FLT, or amino acid tracers ^37, 39^. Anti-angiogenic therapies can lead to overestimation of response based on criteria which measure the dimensions of contrast enhancement, and progression frequently presents as enlarging FLAIR abnormality without enhancement. Anti-angiogenic pseudoresponse confounds the evaluation of efficacy at early time points in treatment. As with pseudoprogression, a range of quantitative MRI ^40^ and PET techniques ^41, 42^ have been evaluated as potential imaging biomarkers of response.

Currently, there are no prospectively validated imaging biomarkers which are reliable discriminators of true response. A multidisciplinary working group to address the complexities of treatment response assessment in neuro-oncology have produced guidelines for current clinical trials (RANO). This group, led by Ben Ellingson, have proposed standardized imaging protocols with tiered complexity to establish standard imaging practices for both clinical management and translational therapeutic protocols ^43^.

Using quantitative imaging techniques, investigators have identified imaging features that are not accessible by visual inspection and which correlate with molecular characteristics of the tumor and biologic behavior ^44, 45^. This application of quantitative imaging using MRI features extracted from imaging data may allow non-invasive assessment of regional heterogeneity, identify the presence of *EGFR* amplification and *IDH* mutation ^46, 47^, differentiate infiltrative tumor from perilesional edema ^48^, differentiate recurrence from radiation treatment effect, and have prognostic significance in patients with newly diagnosed GBM ^44, 46^. Immunotherapy of GBM challenges response evaluation criteria based on enhancement as well, with the immune-mediated inflammation associated with response, mimicking progression. In a report of retrospective analysis of MRI parameters, including DCE, DSC, and arterial spin labeling (ASL), MR spectroscopic measurement of myoinositol and Na23, and FLT PET, the authors suggest that advanced neuroimaging techniques may differentiate immunologic response from tumor progression ^49^.

Elderly patients with GBM constitute an especially refractory challenge. Age over 60 years has been a consistent poor prognostic factor through the modern history of GBM therapeutics. The increasing incidence with age coupled to the aging population demographic means that the management of 70 and 80 year olds with GBM is becoming a numerically more frequent problem. Several trials have compared regimens which shortened the duration of radiation therapy and compared radiation therapy alone to radiation with chemotherapy and even chemotherapy alone, producing similar outcomes ^50^. MGMT promoter methylation status is an important prognostic and predictive marker. For MGMT promoter methylated patients, in one trial, treatment with temozolomide alone was equivalent to short course radiation therapy. Abbreviated courses of radiation appear to be similar in efficacy to a full six weeks of radiation, but none of these trials compared their experimental regimen to the full six-week regimen of chemoradiation followed by six months of adjuvant temozolomide. Although controversy remains about the management of elderly patients, evolving consensus suggests that patients between 60 and 70 should be considered for similar treatment as younger patients while patients over 70 or with significant medical comorbidities be treated with a modified regimen. A randomized prospective trial comparing 4,000 cGy over three weeks versus concurrent chemoradiation to the same total dose followed by six months of adjuvant temozolomide demonstrated a survival benefit to the chemotherapy cohort, with a beneficial effect in both the MGMT methylated and the unmethylated groups, though larger in the MGMT methylated cohort ^51^. This regimen is currently considered by many neuro-oncology centers to be the standard therapy.

## Conclusion

Although the treatment options for patients with GBM are far from satisfactory, and overall survival for patients with newly diagnosed GBM remains short, this is an optimistic time for neuro-oncology. Neuro-oncology centers with multi-disciplinary teams including neurosurgery, neuro-oncology, neuroradiology, and neuropathology are seeing median overall survival increasing, and the number of long-term survivors is increasing as well. The translational research challenges over the next five years include the systematic evaluation of immunotherapy using cell-based treatments and checkpoint inhibitors, elucidating the factors that create sensitivity and resistance to molecularly targeted therapies, and the development of increasingly accurate imaging biomarkers of treatment response. Radiogenomics and other applications of quantitative neuroimaging will improve our ability to identify biologically relevant characteristics of tumors non-invasively. The current generation of clinical trials are incorporating powerful insights into the relationship between molecular genetics and biologic behavior, attention to the issue of drug delivery to tumor and pharmacodynamics, and the interplay of mechanisms of tumor immunity and immunosuppression in the tumor microenvironment.

This review has provided an overview of the rapidly evolving diagnostic, therapeutic, and imaging aspects of the management of patients with GBM. The most important recommendation regarding the care of patients with GBM is the importance of the role of neuro-oncologic centers of excellence with experience in the diagnosis and management of these patients based on high-volume neurosurgical and neuro-oncology practice, as equally important, with access to clinical trials.
